# Tetranectin and Paraoxonase 1 in Patients with Varying Stages of Heart Failure: A Cross-Sectional Analysis

**DOI:** 10.3390/clinpract15050086

**Published:** 2025-04-25

**Authors:** Paula Alexandra Vulciu, Luminita Pilat, Maria-Daniela Mot, Voicu Dascau, Calin Daniel Popa, Norberth-Istvan Varga, Maria Puschita

**Affiliations:** 1 Department of Biochemistry, “Vasile Goldis” Western University, B-dul Revolutiei Nr. 96, 310025 Arad, Romania; vulciu.paula@uvvg.ro (P.A.V.); luminita.pilat@yahoo.com (L.P.); 2 Department of General Medicine, “Vasile Goldis” Western University, B-dul Revolutiei Nr. 96, 310025 Arad, Romania; 3 Department of Obstetrics and Gynecology, Medlife-Genesys Clinic, Dr. Cornel Radu Nr. 3, 310329 Arad, Romania; drdascauvoicu@yahoo.com; 4 Saint Luke of Crimeea Medical Center, B-dul Nicolae Titulescu Nr. 332, 310328 Arad, Romania; calinpopa@ymail.com; 5 Doctoral School, Department of General Medicine, “Victor Babes” University of Medicine and Pharmacy, Eftimie Murgu Square 2, 300041 Timisoara, Romania; norberth.varga@umft.ro; 6 Department of Internal Medicine, “Vasile Goldis” Western University, B-dul Revolutiei Nr. 96, 310025 Arad, Romania; mpuschita.mp@gmail.com

**Keywords:** heart failure, tetranectin, paraoxonase 1, heart failure biomarkers, heart failure severity, echocardiography

## Abstract

**Background:** Heart failure (HF) is a leading cause of mortality across the globe, prompting ongoing research into novel biomarkers for improved risk stratification and patient management. **Methods:** This cross-sectional study aimed to investigate the relationship between two promising biomarkers, tetranectin and paraoxonase 1, and the severity of heart failure in a cohort of 87 patients with cardiovascular risk factors. Participants were categorized into three groups based on their New York Heart Association (NYHA) classification: no HF (Control), NYHA class I (G1), and NYHA class II-IV (G2). **Results:** Our analysis revealed a stepwise decrease in both TETRA and PON1 levels with increasing HF severity, with the Control group exhibiting the highest levels and the G2 group the lowest. Interestingly, a significant positive correlation between TETRA and PON1 was observed only in the Control group, suggesting a potential interplay between these biomarkers in healthy individuals that may be disrupted with the onset of HF. Furthermore, both TETRA and PON1 were positively associated with left ventricular ejection fraction (LVEF) and negatively associated with diastolic dysfunction, indicating their potential involvement in both systolic and diastolic cardiac function. **Conclusions:** These findings suggest that TETRA and PON1 may serve as valuable biomarkers for assessing HF severity and prognosis. Further research is warranted to validate these findings in larger, prospective studies and to explore their clinical utility in guiding treatment decisions.

## 1. Introduction

Heart failure (HF) is a complex clinical syndrome characterized by the inability of the heart to pump sufficient blood to meet the body’s metabolic demands [1,2]. This can result from structural or functional impairments of the heart, leading to a range of symptoms including shortness of breath, fatigue, and fluid retention. HF represents a significant global health burden, affecting approximately 64.3 million people worldwide [3]. In Europe, the prevalence of HF is estimated to be 1–2% of the adult population, with higher rates observed in older age groups [4]. The diagnosis and management of HF rely on a combination of clinical assessment, imaging techniques such as echocardiography, and laboratory biomarkers [5,6]. While established biomarkers like BNP and NT-proBNP excel in diagnosing acute HF via natriuretic pathways, they offer limited insight into oxidative stress and extracellular matrix remodeling—processes central to HF progression where TETRA and PON1 hold unique potential, as evidenced by their dysregulation in related cardiovascular conditions [7,8].

Tetranectin, a plasminogen-binding protein first identified in 1986 for its role in cell adhesion and spreading [9], has garnered increasing attention for its implications in cardiovascular diseases and cancer pathophysiology [10,11,12,13]. While its precise physiological mechanisms are still under research, tetranectin is known to be involved in various processes, including extracellular matrix remodeling, fibrinolysis, and angiogenesis [14]. Studies have reported dysregulation in tetranectin levels in patients with cardiovascular disease, myocardial infarction, neurodegeneration, atherosclerosis, diabetes, sepsis, and certain cancers [10,11,12,14,15]. In the context of heart failure, tetranectin has emerged as a potential biomarker, with preliminary evidence suggesting that its levels may correlate with disease severity and prognosis [16,17,18].

Paraoxonase 1 (PON1) is an enzyme associated with high-density lipoprotein (HDL) cholesterol, initially discovered in the 1950s for its ability to hydrolyze organophosphates [19]. While its name originates from its capacity to detoxify the insecticide paraoxon, PON1 has since been recognized for its multifaceted role in cardiovascular health. This enzyme exhibits antioxidant properties, protecting low-density lipoprotein (LDL) cholesterol from oxidation, a crucial step in the development of atherosclerosis [20,21,22]. Furthermore, PON1 is involved in innate immunity and the modulation of inflammation, both of which are implicated in the pathogenesis of heart failure [22,23]. Given its protective effects against oxidative stress and inflammation, PON1 has been investigated as a potential biomarker for various cardiovascular diseases, including coronary artery disease and heart failure [24,25].

This study aims to investigate the relationship between tetranectin (TETRA) and paraoxonase 1 (PON1) levels and the severity of heart failure in patients with hypertension and dyslipidemia. Specifically, we sought to (1) compare TETRA and PON1 levels across different stages of heart failure severity, (2) assess the correlation between TETRA and PON1 levels within each group, and (3) explore the association of TETRA and PON1 with echocardiographic parameters. We hypothesized that TETRA and PON1 levels decrease with increasing HF severity and correlate with echocardiographic indices of systolic and diastolic function, aiming to elucidate their prognostic potential.

## 2. Materials and Methods

### 2.1. Study Design and Population

This cross-sectional study included adult patients (age > 18 years) with cardiovascular risk factors, such as hypertension, dyslipidemia, diabetes, and/or obesity, who were recruited from “Centrul Medical Sf. Luca al Crimeei” in Arad, Romania, between 1 January and 1st August 2024. Recruitment occurred during scheduled appointments, where eligible patients were approached by study staff, informed about the research objectives, and invited to participate. Inclusion criteria required (1) regular attendance at the clinic for period check-ups (at least one visit in the prior six months); (2) the presence of one or more cardiovascular risk factors (diabetes, obesity, smoking, hypercholesterolemia, kidney disease, etc.); and (3) written informed consent. Exclusion criteria included (1) refusal to consent; (2) acute cardiovascular events within the past 30 days; and (3) the presence of active malignancy or severe hepatic or renal dysfunction (cirrhosis, end-stage renal disease), as these conditions could confound biomarker levels or echocardiographic findings. Patients were not excluded based on stable chronic medications (e.g., antihypertensives, statins, etc.) or lifestyle factors (e.g., diet, physical activity).

To investigate the relationship between TETRA and PON1 levels and heart failure severity, the study population was divided into three groups based on their New York Heart Association (NYHA) heart failure classification: patients with no heart failure (Control group), patients with NYHA class I heart failure (G1 group), and patients with NYHA class II-IV heart failure (G2 group). Patients’ NYHA categories were assessed by experienced cardiologists (P.A.V., C.D.P.), consistent with ESC guidelines, and prior medical records were verified. This stratification allowed for the examination of potential differences in TETRA and PON1 levels across varying degrees of heart failure severity.

### 2.2. Data Collection

Data were collected prospectively during clinic visits and ensured a standardized approach across all participants. Demographic information (age, sex, and smoking status) was obtained via patient interviews. Clinical characteristics were gathered through a physical examination and medical record review. Upon each visit, body mass index (BMI) and blood pressure were measured. Lipid profiles (total cholesterol and HDL-cholesterol were measured), and diabetes status (defined by prior diagnosis or HbA1c > 6.5%) were extracted from fasting blood samples collected on the same day as the visit and analyzed at the clinic’s certified laboratory using standard enzymatic assays (Roche Cobas 6000 analyzer). Echocardiographic parameters were assessed by two experienced cardiologists (P.A.V. or C.D.P.) using a Philips EPIQ 7 ultrasound system with a 3D X5-1 transducer. Measurements included 3D left ventricular ejection fraction (LVEF), global longitudinal strain (GLS), left atrial reservoir strain (LAS), systolic function, and diastolic dysfunction. Plasma TETRA and PON1 levels were quantified via a commercially available enzyme-linked immunosorbent assay (ELISA) kit (Human Tetranectin ELISA, MyBioSource, San Diego, CA, USA), and via a colorimetric assay (Paraoxonase 1 Activity Assay Kit, Abcam, Cambridge, UK), respectively. Venous blood samples (5 mL) were drawn into EDTA tubes after confirming that the patients had fasted overnight. Diastolic dysfunction was assessed using Philips EPIQ 7 echocardiography, graded from 0 to III based on American Society of Echocardiography (ASE) guidelines, incorporating parameters such as E/A ratio, E/e’ ratio, and left atrial volume index.

### 2.3. Ethical Considerations

This study followed the principles of the Helsinki Declaration on Medical Protocol and Ethics. Ethical approval was obtained from the Ethics Committee of Centrul Medical Sf. Luca al Crimeei, Romania, reference number 39/28.12.2023, approval date: 28 December 2023 and the “Vasile Goldis” Western University of Arad, Romania, reference number 75/29.04.2024, approval date: 29 April 2024. All patients provided written informed consent before enrollment in the study.

### 2.4. Statistical Analysis

Continuous variables were assessed for normality using the Shapiro–Wilk test. Differences in baseline characteristics between the three groups (Control, Group 1, and Group 2) were assessed using one-way ANOVA for normally distributed variables, the Kruskal–Wallis test for non-normally distributed variables, and the chi-square test for categorical variables. Pairwise comparisons for TETRA and PON1 levels were performed using the Mann–Whitney U test with a Bonferroni correction. Correlations between TETRA and PON1 were assessed using Spearman’s rank correlation for non-normally distributed data and Pearson’s correlation for normally distributed data. Multiple linear regression analyses were conducted to examine the associations between TETRA/PON1 levels and echocardiographic parameters. To assess the independent associations of TETRA and PON1 with HF severity, ordinal logistic regression was performed, adjusting for age, sex, diabetes status, and LVEF as covariates. Data were analyzed using SPSS version 26 (IBM Corp., Armonk, NY, USA). Statistical significance was set at *p* < 0.05.

## 3. Results

### 3.1. General Characteristics of the Study Population

This study included 87 patients who consented to participate between January and August 2024. This cohort was categorized into three groups based on heart failure severity: Control (no heart failure, *n =* 20), G1 (NYHA class I, *n =* 30), and G2 (NYHA class II-IV, *n =* 37). The mean age of the participants was 56.56 years (SD = 13.82), with 65.9% (*n =* 54) being female. The overall body mass index (BMI) was 30.19 kg/m^2^ (SD = 5.88). 48.8% (*n =* 40) of the patients had type II diabetes. Most patients (74.4%, *n =* 61) reported not smoking. The mean values for total cholesterol and HDL-cholesterol in the overall study population were 249.66 mg/dL (SD = 47.25) and 36.96 mg/dL (SD = 9.21), respectively. Echocardiographic assessments revealed a mean left ventricular ejection fraction (LVEF) of 54.29% (SD = 5.36), a mean global longitudinal strain (GLS) of −18.22% (SD = 2.99), and an average left atrial strain as reservoir (LAS) of 32.16% (SD = 10.47). Mean TETRA and PON1 levels were 7.34 mg/L (SD = 720.96) and 21.28 U/L (SD = 22.89), respectively.

Prior to conducting statistical comparisons, the distributions of all continuous variables were assessed for normality using the Shapiro–Wilk test. Based on these tests, age (*p* = 0.633) and BMI (*p* = 0.169) were determined to be normally distributed, while total cholesterol (*p* = 0.01), GLS (*p* = 0.01), LAS (*p* < 0.01), FEVS 3D (*p* < 0.01), TETRA (*p* < 0.01), and PON1 (*p* = 0.0.1) were not. This information guided the selection of appropriate statistical tests for subsequent analyses.

Table 1 presents the baseline characteristics of the study population stratified by heart failure severity. Significant differences between the groups were observed for age (*p* < 0.01), BMI (*p* = 0.015), the presence of diabetes (*p* < 0.01), GLS (*p* = 0.01), LAS (*p* < 0.01), LVEF (*p* < 0.01), TETRA (*p* < 0.01), and PON1 (*p* < 0.01)]. These differences highlight the varying clinical profiles of the three groups and underscore the potential influence of heart failure severity on these parameters.

### 3.2. Correlation Between TETRA and PON1 Across Study Groups

To assess differences in TETRA and PON1 levels across the three groups (Control, G1, and G2), the Kruskal–Wallis test was employed. This test was chosen because the distributions of TETRA and PON1 were not uniformly normal across all groups. TETRA levels were normally distributed in the G1 group (*p* = 0.898) and the G2 group (*p* = 0.230), but not in the Control group (*p* = 0.016). Conversely, PON1 levels were normally distributed only in the Control group (*p* = 0.357), while demonstrating non-normal distributions in both G1 and G2 groups (*p* = 0.014 and 0.013, respectively). Table 2 summarizes these findings. The analysis revealed statistically significant differences in both TETRA (H = 63.513, *p* < 0.001) and PON1 (H = 60.051, *p* < 0.001) levels across the groups. This indicates that the median levels of both biomarkers are not equal across the three groups, suggesting a potential association between TETRA and PON1 levels and the severity of heart failure.

Pairwise comparisons using the Mann–Whitney U test with a Bonferroni correction (adjusted alpha = 0.0167) were conducted to determine the specific group differences in TETRA levels. The analysis revealed a clear stepwise reduction in TETRA levels with increasing heart failure severity. The Control group (no heart failure) exhibited significantly higher TETRA levels compared to both the G1 group (NYHA class I heart failure; U = 0.000, *p* < 0.001) and the G2 group (NYHA class II–IV heart failure; U = 0.000, *p* < 0.001). Furthermore, the G1 group also showed significantly higher TETRA levels than the G2 group (U = 74.000, *p* < 0.001). These findings strongly suggest a negative association between TETRA levels and heart failure severity.

Similarly to TETRA, pairwise comparisons using the Mann–Whitney U test with a Bonferroni correction (adjusted alpha = 0.0167) revealed a stepwise decrease in PON1 levels with increasing heart failure severity. The Control group demonstrated significantly higher PON1 levels compared to both the G1 group (U = 2.000, *p* < 0.001) and the G2 group (U = 0.000, *p* < 0.001). Additionally, the G1 group exhibited significantly higher PON1 levels than the G2 group (U = 111.000, *p* < 0.001). These results indicate a strong negative association between PON1 levels and heart failure severity, mirroring the pattern observed for TETRA.

To quantify the magnitude of differences, effect sizes were calculated as Cohen’s d for pairwise comparisons. Table 3 presents the results. For TETRA, we observed large effects: Control vs. G1 (d = 2.43), Control vs. G2 (d = 3.19), and G1 vs. G2 (d = 1.96). Similarly, PON1 showed substantial effects: Control vs. G1 (d = 3.59), Control vs. G2 (d = 4.42), and G1 vs. G2 (d = 1.67). These large effect sizes underscore the pronounced stepwise decline in both biomarkers with increasing HF severity.

Correlation analysis revealed a significant positive correlation between TETRA and PON1 levels in the Control group (Spearman’s rho = 0.671, *p* = 0.001), indicating that higher TETRA levels were associated with higher PON1 levels in this group. However, no significant correlations were observed in either the G1 group (Spearman’s rho = 0.123, *p* = 0.526) or the G2 group (Pearson’s r = 0.281, *p* = 0.113). These findings suggest that the relationship between TETRA and PON1 may differ depending on the presence and severity of heart failure, with a strong association observed only in patients without heart failure. Table 4 summarizes these findings.

### 3.3. Association of TETRA and PON1 with Echocardiographic Parameters

To further explore the relationship between the biomarkers and cardiac function, multiple linear regression analyses were performed with TETRA and PON1 as outcome variables, using echocardiographic parameters as predictors (Table 5). Both regression models were statistically significant (TETRA: F(5, 76) = 28.238, *p* < 0.001, R = 0.806, R-squared = 0.650; PON1: F(5, 76) = 24.241, *p* < 0.001, R = 0.784, R-squared = 0.615), explaining 65% and 61.5% of the variance in TETRA and PON1 levels, respectively. For TETRA, LVEF (B = 39.547, β = 0.294, *p* = 0.003) and diastolic dysfunction (B = −1045.523, β = −0.549, *p* < 0.001) were significant independent predictors. Higher LVEF values were associated with higher TETRA levels, while the presence of diastolic dysfunction was associated with lower TETRA. For PON1, GLS (B = 1.993, β = 0.260, *p* = 0.007), LVEF (B = 1.006, β = 0.236, *p* = 0.022), and diastolic dysfunction (B = −27.137, β = −0.449, *p* < 0.001) were significant predictors. Higher GLS and LVEF values were associated with higher PON1, while diastolic dysfunction was associated with lower PON1. These findings suggest that both TETRA and PON1 may be related to systolic and diastolic cardiac function. The positive associations with LVEF and GLS could indicate that these biomarkers play a role in maintaining optimal left ventricular contractility, while the negative associations with diastolic dysfunction might suggest that lower TETRA and PON1 levels are involved in its pathophysiology.

### 3.4. Multivariate Analysis of Tetranectin and Paraoxonase 1 as Predictors of Heart Failure Severity

To further elucidate the roles of TETRA and PON1 in heart failure (HF) progression, a multivariate analysis was conducted to assess their independent associations with HF severity, adjusting for key clinical and echocardiographic confounders. Given the stepwise decline in TETRA and PON1 levels with worsening HF observed in our earlier analyses, we sought to determine whether these biomarkers retained predictive value beyond established risk factors. An ordinal logistic regression model was employed, with HF severity (Control, G1, G2) as the ordinal outcome variable. Predictor variables included TETRA, PON1, age, sex, diabetes status, and left ventricular ejection fraction (LVEF), selected based on their significant differences across groups (Table 1) and their established evidence from the HF literature. Specifically, TETRA and PON1 were included as the primary biomarkers of interest due to their significant stepwise reductions across HF groups (see Section 3.2) and their associations with echocardiographic parameters (see Section 3.3). Age was incorporated because it differed markedly across our groups (Table 1, *p* < 0.01) and is a recognized driver of HF risk and severity. Sex was added as a standard demographic covariate, given its reported influence on HF epidemiology and potential modulation of biomarker expression. Diabetes status was included due to its significant prevalence differences across groups (Table 1, *p* < 0.01) and its established role as a comorbidity that exacerbates HF through metabolic and vascular pathways, which may intersect with PON1’s antioxidant functions. Finally, LVEF was selected as a direct measure of systolic function, showing a clear decline with HF severity (Table 1, *p* < 0.01) and a positive association with both biomarkers (see Section 3.3), making it a critical confounder to adjust for in evaluating TETRA and PON1’s independent effects. Additional confounders (e.g., antihypertensive or lipid-lowering medications, smoking) were not adjusted due to sample size constraints.

The model was statistically significant (χ^2^(6) = 72.341, *p* < 0.001), explaining approximately 68% of the variance in HF severity (Nagelkerke R^2^ = 0.679). Table 6 presents the results. TETRA emerged as a significant independent predictor of HF severity (OR = 0.998, 95% CI: 0.997–0.999, *p* = 0.002), suggesting that for each 1 mg/L increase in TETRA, the odds of being in a higher HF severity category decreased by 0.2%, after adjusting for other variables. Similarly, PON1 was independently associated with HF severity (OR = 0.941, 95% CI: 0.908–0.975, *p* = 0.001), indicating that a 1 U/L increase in PON1 reduced the odds of worse HF by 5.9%. Age (OR = 1.074, 95% CI: 1.041–1.108, *p* < 0.001) and diabetes (OR = 2.312, 95% CI: 1.145–4.667, *p* = 0.019) also significantly predicted higher HF severity, consistent with their known roles as HF risk factors. LVEF showed a protective effect (OR = 0.892, 95% CI: 0.841–0.946, *p* < 0.001), where higher LVEF values were linked to lower HF severity odds. Sex was not a significant predictor (OR = 1.214, 95% CI: 0.612–2.408, *p* = 0.581).

## 4. Discussion

This study aimed to investigate the relationship between tetranectin (TETRA) and paraoxonase 1 (PON1) levels and the severity of heart failure in patients with hypertension and dyslipidemia. Our findings reveal a compelling link between these biomarkers and the progression of heart failure.

Firstly, we found that both TETRA and PON1 levels decrease in a stepwise manner as heart failure worsens. This means that patients with no heart failure had the highest levels of these biomarkers, while those with more severe heart failure (NYHA class II-IV) had the lowest levels. This pattern was consistent for both TETRA and PON1, suggesting a parallel decline in their levels with disease progression. Secondly, we observed a strong positive correlation between TETRA and PON1 levels, but only in patients without heart failure. This suggests that in healthy individuals, these two biomarkers might be working together or influenced by similar factors. However, this relationship seems to disappear as heart failure develops, indicating a potential disruption in their interplay. Finally, our analysis revealed that TETRA and PON1 levels are associated with specific measures of cardiac function. Both biomarkers were positively associated with LVEF, a measure of the heart’s pumping ability, suggesting their potential role in maintaining efficient heart contraction. Conversely, both were negatively associated with diastolic dysfunction, indicating that lower levels of these biomarkers might be linked to impaired heart relaxation. These findings provide valuable insights into the potential roles of TETRA and PON1 in the context of heart failure. The consistent decline in their levels with disease progression, along with their associations with specific cardiac function parameters, suggests that these biomarkers might be involved in the underlying mechanisms of heart failure development. The loss of correlation between TETRA and PON1 in HF patients (G1, G2) versus controls may reflect a disease-driven uncoupling of their shared pathways, such as oxidative stress modulation (PON1) and matrix remodeling (TETRA), suggesting HF alters their physiological interplay.

Our findings contribute to a growing body of evidence supporting the roles of tetranectin and paraoxonase 1 in heart failure. The observed decrease in both TETRA and PON1 levels with increasing heart failure severity aligns with previous reports. Chen et al. (2015) demonstrated decreased serum tetranectin levels in patients with stable coronary artery disease [16], while McDonald et al. (2020) reported reduced circulating tetranectin levels in heart failure patients and highlighted its potential as a diagnostic biomarker [17]. Similarly, Hammadah (2017) found that reduced baseline PON1 activity and decreased levels over time were associated with adverse outcomes in heart failure patients, supporting our observation of lower PON1 levels in those with more severe heart failure [26]. The stepwise decline in TETRA may stem from impaired fibrinolysis or increased myocardial fibrosis, as suggested by its myocardial expression [17], while PON1’s reduction likely reflects heightened oxidative stress overwhelming its antioxidant capacity [19].

The associations we found between these biomarkers and echocardiographic parameters are consistent with studies suggesting their involvement in cardiac function. Connelly et al. (2021) observed an inverse correlation between changes in PON1 mass and changes in left ventricular volume after kidney transplantation, aligning with our finding that higher PON1 levels were associated with better LVEF [27]. Grzegorzewska et al. (2021) further reinforced the cardiovascular protective role of PON1, reporting an association between lower PON1 activity and a higher prevalence of atherogenic dyslipidemia and cardiovascular mortality in hemodialysis patients [28]. Our Control group TETRA levels (17.92 ± 7.97 mg/L) align with the upper range of healthy values reported in the literature (e.g., 11.18 ± 2.20 mg/L, Chen et al., 2015 [16]; 10–12 mg/L), while PON1 levels (57.37 ± 19.15 U/L) match typical healthy norms (~50–150 U/L, Mackness et al., 2004 [25]). G2 levels (TETRA 2.70 ± 1.04 mg/L, PON1 7.16 ± 1.98 U/L) reflect reductions consistent with heart failure, as observed in McDonald et al. (2020) [17] for TETRA and Hammadah et al. (2017) [26] for PON1 (CHF range 52–111 U/L), supporting their relevance across HF severity and enhancing generalizability despite our single-center cohort.

Ikeda et al. (1998) found that serum PON1 activity was significantly lower in patients with non-insulin-dependent diabetes mellitus, highlighting the potential interplay between PON1, diabetes, and heart failure [29]. Given their association with diastolic dysfunction, TETRA and PON1 could enhance early detection of HF or monitor disease progression alongside BNP, though their integration into routine practice requires prospective validation.

Our multivariate analysis further strengthens the case for TETRA and PON1 as potential biomarkers in heart failure, revealing their independent associations with HF severity even after adjusting for age, sex, diabetes, and LVEF. The finding that higher levels of both TETRA and PON1 correspond to reduced odds of worse HF severity (OR = 0.998 and 0.941, respectively) aligns with their stepwise decline across our study groups and echoes prior reports of their protective roles in cardiovascular health. For instance, McDonald et al. (2020) noted reduced TETRA levels in HF patients and suggested its diagnostic potential [17], while Hammadah et al. (2017) linked lower PON1 activity to adverse HF outcomes [26]. Intriguingly, the persistence of these associations despite the inclusion of LVEF, a robust indicator of systolic function, suggests that TETRA and PON1 may capture aspects of HF progression beyond mere pump efficiency, possibly reflecting processes like fibrosis or oxidative stress, as hinted by their ties to diastolic dysfunction in our regression models. This independence from traditional risk factors like age and diabetes, which also emerged as significant predictors in our model, underscores their potential as complementary tools for risk stratification, warranting further exploration in longitudinal settings to confirm their prognostic value.

This study has several limitations that should be considered when interpreting the results. Firstly, the cross-sectional design precludes the establishment of causality between TETRA/PON1 levels and HF severity. While we observed associations, longitudinal studies are needed to determine if these biomarkers can predict HF progression. Secondly, our study population consisted solely of patients with hypertension and dyslipidemia from a single medical center in Romania, potentially limiting the generalizability of our results to other HF populations. Thirdly, the relatively small sample size, particularly after stratification into three groups, may have limited the statistical power to detect subtle differences. Sensitivity analyses to test the robustness of non-parametric findings were not performed, potentially limiting confidence in the results’ stability across subgroups. Clinic-based recruitment may introduce selection bias toward patients with more advanced disease, and the cross-sectional design prevents tracking TETRA and PON1 changes over time, both of which limit causal inference. Finally, while we collected data on several clinical parameters, the analyses did not explicitly adjust for all potential confounders, such as specific medication use or other comorbidities, which could influence both biomarker levels and HF severity. Future studies with larger, more diverse populations and comprehensive adjustments for confounding factors are warranted to validate and expand upon our findings. Furthermore, future studies, including animal models knocking out or reducing TETRA and PON1, could elucidate their causal roles in HF development and inform underlying mechanisms.

## 5. Conclusions

To conclude, this study demonstrated a compelling association between decreasing levels of TETRA and PON1 and increasing severity of heart failure in patients with hypertension and dyslipidemia. Both biomarkers were independently associated with echocardiographic parameters of systolic and diastolic function, suggesting their potential role in the pathophysiology of HF. These findings highlight the potential of TETRA and PON1 as valuable biomarkers for risk stratification and disease monitoring in HF. However, further research is needed to validate these findings in larger, more diverse populations and to establish their clinical utility in guiding treatment decisions.

## Figures and Tables

**Table 1:** Baseline characteristics of patients stratified by heart failure severity. SD = standard deviation; BMI = body mass index; LAS = left atrial strain; GLS = global longitudinal strain; LVEF = left ventricular ejection fraction; TETRA = tetranectin; PON1 = paraoxonase 1.

Variable	Overall Population (*n =* 87)	Control Group (*n =* 20)	Group 1 (*n =* 30)	Group 2 (*n =* 37)	Statistical Test	*p*-Value
Age (years), mean (SD)	56.56 (13.82)	39 (5.45)	60.28 (11.91)	63.94 (8.89)	ANOVA	<0.01
Female sex, *n* (%)	54 (65.9)	14 (70)	16 (55.2)	24 (72.7)	Chi-square	0.314
Smoking status, *n* (%)	21 (25.6)	5 (25)	7 (24.1)	9 (27.3)	Chi-square	0.958
BMI (kg/m^2^), mean (SD)	30.19 (5.88)	28.13 (5.21)	32.62 (6.42)	29.31 (5.15)	ANOVA	0.015
Total cholesterol (mg/dL), mean (SD)	249.66 (47.25)	253.8 (62.27)	238.28 (47.07)	257.15 (35)	Kruskal–Wallis	0.09
Diabetes, *n* (%)	40 (48.8)	9 (45)	20 (66.67)	11 (29.72)	Chi-square	<0.01
GLS (%), mean (SD)	−18.22 (2.99)	−21.8 (1.67)	−17.62 (2.71)	−16.58 (1.83)	Kruskal–Wallis	0.147
LAS (%), mean (SD)	32.16 (10.47)	41.3 (5.64)	35.17 (10.85)	23.97 (5.12)	Kruskal–Wallis	<0.01
LVEF (%), mean (SD)	54.29 (5.36)	59 (4.32)	56.03 (4.7)	49.91 (2.44)	Kruskal–Wallis	<0.01
TETRA (mg/L), mean (SD)	7.34 (7.20)	17.92 (7.97)	5.34 (1.64)	2.70 (1.04)	Kruskal–Wallis	<0.01
PON1 (U/L), mean (SD)	21.28 (22.89)	57.37 (19.15)	12.47 (4.21)	7.16 (1.98)	Kruskal–Wallis	<0.01

**Table 2:** Normality testing for TETRA and PON1 levels by group. TETRA = tetranectin; PON1 = paraoxonase 1.

Biomarker	Group	Shapiro–Wilk Statistic	df	*p*-Value
TETRA	Control	0.877	20	0.016
	G1	0.983	29	0.898
	G2	0.958	33	0.23
PON1	Control	0.949	20	0.357
	G1	0.907	29	0.014
	G2	0.914	33	0.013

**Table 3:** Effect sizes (Cohen’s d) for pairwise comparisons of TETRA and PON1 levels across study groups.

Comparison	TETRA (Cohen’s d)	PON1 (Cohen’s d)
Control vs. G1	2.43	3.59
Control vs. G2	3.19	4.42
G1 vs. G2	1.96	1.67

**Table 4:** Correlation between TETRA and PON1 levels across study groups.

Group	Correlation Coefficient	*p*-Value
Control	0.671	0.001
G1	0.123	0.526
G2	0.281	0.113

**Table 5:** Linear regression results: association of echocardiographic parameters with TETRA and PON1 levels. B = unstandardized regression coefficient; β = standardized regression coefficient; GLS = global longitudinal strain; LAS = left atrial strain; LVEF = left ventricular ejection fraction; TETRA = tetranectin; PON1 = paraoxonase 1.

Biomarker	Predictor Variable	B	β	*p*-Value
TETRA	GLS	35.705	0.148	0.099
	LAS	−1.014	−0.015	0.878
	LVEF	39.547	0.294	0.003
	Systolic function	134.065	0.049	0.51
	Diastolic dysfunction	−1045.523	−0.549	<0.001
PON1	GLS	1.993	0.26	0.007
	LAS	0.038	0.018	0.861
	LVEF	1.006	0.236	0.022
	Systolic function	−1.857	−0.021	0.784
	Diastolic dysfunction	−27.137	−0.449	<0.001

**Table 6:** Ordinal logistic regression results. OR = odds ratio; CI = confidence interval; TETRA = tetranectin; PON1 = paraoxonase 1; LVEF = Left ventricular ejection fraction.

Predictor Variable	OR	95% CI	*p*-Value
TETRA (mg/L)	0.998	0.997–0.999	0.002
PON 1 (U/L)	0.941	0.908–0.975	0.001
Age (years)	1.074	1.041–1.108	<0.001
Sex (female vs. male)	1.214	0.612–2.408	0.581
Diabetes (yes vs. no)	2.312	1.145–4.667	0.019
LVEF (%)	0.892	0.841–0.946	<0.001

## Data Availability

Data are available upon request from the corresponding author.

## Abbreviations

The following abbreviations are used in this manuscript:

HF	Heart Failure
TETRA	Tetranectin
PON1	Paraoxonase 1
NYHA	New York Heart Association
GLS	Global Longitudinal Stain
LAS	Left Atrial Strain
BMI	Body Mass Index
LVEF	Left Ventricular Ejection Fraction
SD	Standard Deviation
